# Serum CCL2 Is a Prognostic Biomarker for Non-Metastatic Castration-Sensitive Prostate Cancer

**DOI:** 10.3390/biomedicines10102369

**Published:** 2022-09-22

**Authors:** Hiroaki Iwamoto, Kouji Izumi, Ryunosuke Nakagawa, Ren Toriumi, Shuhei Aoyama, Taiki Kamijima, Takafumi Shimada, Hiroshi Kano, Tomoyuki Makino, Renato Naito, Suguru Kadomoto, Hiroshi Yaegashi, Shohei Kawaguchi, Takahiro Nohara, Kazuyoshi Shigehara, Yoshifumi Kadono, Atsushi Mizokami

**Affiliations:** 1 Department of Integrative Cancer Therapy and Urology, Kanazawa University Graduate School of Medical Science, Kanazawa 920-8641, Japan; 2 Department of Urology, Ishikawa Prefectural Central Hospital, Kanazawa 920-8530, Japan

**Keywords:** CCL2, biomarker, prostate cancer, survival, prognostic, chemokine

## Abstract

Purpose: Prostate-specific antigen (PSA) is a useful prostate cancer (PC) biomarker, but some cases reported that PSA does not correlate with the Gleason score. Serum chemokine (CC motif) ligand 2 (CCL2) has been reported to be a potential complementary PSA biomarker, but it remains unclear whether it can be applied to non-metastatic castration-sensitive prostate cancer (nmCSPC) or each section of the stages. Serum CCL2′s usefulness was investigated as a prognostic nmCSPC biomarker in this study. Methods: Serum samples were collected from 379 patients who underwent prostate biopsy at Kanazawa University Hospital from 2007 to 2013. A total of 230 patients with nmCSPC were included in this study of the 255 patients with histologically diagnosed prostate cancer. The serum CCL2 efficacy as a prognostic nmCSPC biomarker was investigated retrospectively. Results: An independent significant predictor of worse OS was CCL2 ≥ 280 pg/dL and CRP ≥ 0.5 mg/dL in multivariate analysis. Gleason score ≥ 8 and CCL2 ≥ 280 pg/dL were independent significant predictors of CRPC-free survival (CFS) worsening in multivariate analysis. Serum CCL2 was a predictive biomarker for OS and CFS in nmCSPC. Furthermore, CCL2 ≥ 280 pg/mL patients had significantly worse visceral metastasis-free survival than those with CCL2 < 280 pg/mL. Conclusion: This study is the first to demonstrate serum CCL2 utility as a biomarker to predict OS and CFS in nmCSPC.

## 1. Introduction

Prostate cancer (PC) is the most common male cancer and the leading cause of cancer-related death in developed countries [1]. Approximately 10% of prostate-cancer patients have distant metastases at diagnosis and the remaining 90% are diagnosed with non-metastatic castration-sensitive prostate cancer (nmCSPC) [2,3]. The prognosis is significantly worse for high-risk PC than for low- and intermediate-risk PCs, with 15-year PC-specific mortality rates ranging 22–38%, while nmCSPC generally has a good prognosis [4,5,6,7]. The high-risk PC (including locally advanced PC) definition, the poor prognosis group in nmCSPC, varies slightly among the guidelines. High risk is defined by the D’Amico risk classification, the European Association of Urology (EAU) guidelines, and the European Society of Medical Oncology (ESMO) guidelines as clinical stage T2c, prostate-specific antigen (PSA) level of 20 ng/mL, and Gleason score (GS) of 8–10, while high risk is defined by the National Comprehensive Cancer Network (NCCN) guidelines as clinical stage T3 or higher [8,9,10,11]. The EAU guidelines classify locally advanced PC with clinical stage T3 or higher or clinical stage N1 as very high risk, while the NCCN guidelines classify clinical stage T3b or higher or primary Gleason pattern 5 or grade group 4 or 5 and core 4 or higher as very high risk [9,11].

PSA is reliable and useful as the only serum biomarker employed for PC risk classification, but it has significant limitations [12,13,14,15]. Some low PSA cases have been reported to also include very poor prognosis cases, including neuroendocrine prostate cancer (NEPC). PSA may not be prognostically relevant when it is higher than a certain level [12,13,14,15]. In prostate cancer, CCL2 has been reported to directly induce proliferation and migration of PC cell lines via activation of phosphoinostitide-3-kinase (PI3K)/serine-threonine kinase (Akt) signaling [16].This led us to consider CCL2 as a potentially useful PC biomarker. Serum chemokine (CC motif) ligand 2 (CCL2) was previously measured in 379 patients (255 had PC) undergoing prostate biopsy and it was found that CCL2 may be a novel biomarker to predict OS in PC, including metastatic cases [17,18]. Serum CCL2 in nmCSPC utility has never been reported. Serum CCL2 was investigated in this study to find whether it was a valid long-term progression and prognosis biomarker of nmCSPC.

## 2. Materials and Methods

Serum samples were collected at the time of prostate biopsy from 379 patients who underwent prostate biopsy at the Kanazawa University Hospital from 2007 to 2013. Venous blood was collected in a blood-collection tube containing a serum-separating medium. After the blood sample had coagulated, it was centrifuged to separate the serum. Serum values of each biomarker were measured using commercially available kits (PSA (Beckman Coulter), CCL2 (R&D Systems)) according to the suppliers’ instruction manuals. A total of 255 out of 379 patients were histologically diagnosed with PC. In the current study, 230 patients diagnosed with nmCSPC were selected for inclusion. All patient medical records, including CRP values, were retrospectively reviewed and analyzed for relevant data. We determined the clinical stage based on the 2017 TNM malignancy classification, eighth edition [19]. Surgical castration, monotherapy with luteinizing hormone-releasing hormone (LH-RH) analogs or antagonists, and combination therapy with antiandrogens and LH-RH analogs or antagonists were included in androgen deprivation therapy (ADT). We defined PSA failure after ADT as an elevated PSA level of at least 2.0 ng/mL and a 25% increase from the nadir, confirmed by a second PSA test at least four weeks later. The diagnosis of castration-resistant prostate cancer (CRPC) was made when the above criteria were met. Each attending physician determined all treatment strategies, blood tests, and imaging intervals after nmCSPC diagnosis. Follow-up for this study ended on 31 January 2022. 

The median follow-up period was 113.0 months. The χ2, Fisher’s exact test, and Mann–Whitney U test were used where appropriate to compare differences in patient characteristics. Overall survival (OS), PC-specific survival (PCSS), CRPC-free survival (CFS), metastasis-free survival (MFS), and visceral metastasis-free survival (VMFS) were estimated using the Kaplan–Meier method, and survival distributions were compared using log-rank tests. We performed multivariate analysis using the Cox proportional-hazards model. The commercially available SPSS software, version 25.0 (SPSSInc., Chicago, IL, USA), and Prism v.9 (GraphPad, SanDiego, CA, USA) were used to perform statistical analyses. In all analyses, a *p*-value < 0.05 indicated statistical significance. The Institutional Review Board of Kanazawa University Hospital (2013–064) approved this study.

## 3. Results

### 3.1. Patients’ Characteristics

Table 1 shows the characteristics of the 230 nmCSPC patients included in this study. The median age at nmCSPC diagnosis was 69 (range = 50–89) years, the median PSA was 9.4 (1.5–227.8) ng/mL, and the median serum CCL2 level was 244.5 (95.3–749) pg/mL. In the study cohort, 74 patients had a GS of 8 or higher, 42 had T3 or higher, and 12 had lymph node metastasis. A total of 12 patients eventually progressed to CRPC and six to mCRPC. All-cause deaths during follow-up were 34 patients and PC-specific deaths were six patients.

### 3.2. Univariate and Multivariate Analyses of OS

We performed a multivariate analysis using the Cox proportional-hazards model to determine whether CCL2 was a useful prognostic factor for nmCSPC. Results of both univariate and multivariate analyses of prognostic factors predicting OS are shown in Table 2. Univariate analysis showed that the significant independent predictors of worse OS were N1 (hazard ratio [HR] = 3.49; *p* = 0.01), PSA ≥ 20 ng/mL (HR = 2.25; *p* = 0.03), GS ≥ 8 (HR = 2.07; *p* = 0.03), CCL2 ≥ 280 pg/dL (HR = 2.07; *p* = 0.04), and CRP ≥ 0.5 mg/dL (HR = 5.30; *p* < 0.001). Independent significant predictors of worse OS were CCL2 ≥ 280 pg/dL (HR = 2.31; *p* = 0.03) and CRP ≥ 0.5 mg/dL (HR = 4.89; *p* < 0.001) in the multivariate analyses. Multivariate analysis was not performed for PCSS due to the very small number of PC-specific deaths (six patients). 

### 3.3. Univariate and Multivariate Analyses of CFS

Prognostic factors predicting CFS were examined in both univariate and multivariate analyses. The results are shown in Table 3. Significant independent predictors of worse CFS were N1 (HR = 6.19; *p* = 0.002), GS ≥ 8 (HR = 13.43; *p* = 0.01) and CCL2 ≥ 280 pg/dL (HR = 6.21; *p* = 0.003) in univariate analysis. Independent significant predictors of worse CFS were GS ≥ 8 (HR = 8.54; *p* = 0.047) and CCL2 ≥ 280 pg/dL (HR = 4.50; *p* = 0.03) in the multivariate analyses.

### 3.4. Kaplan–Meier Curves of OS, PCSS, and CFS in nmCSPC

**Figure 1:**
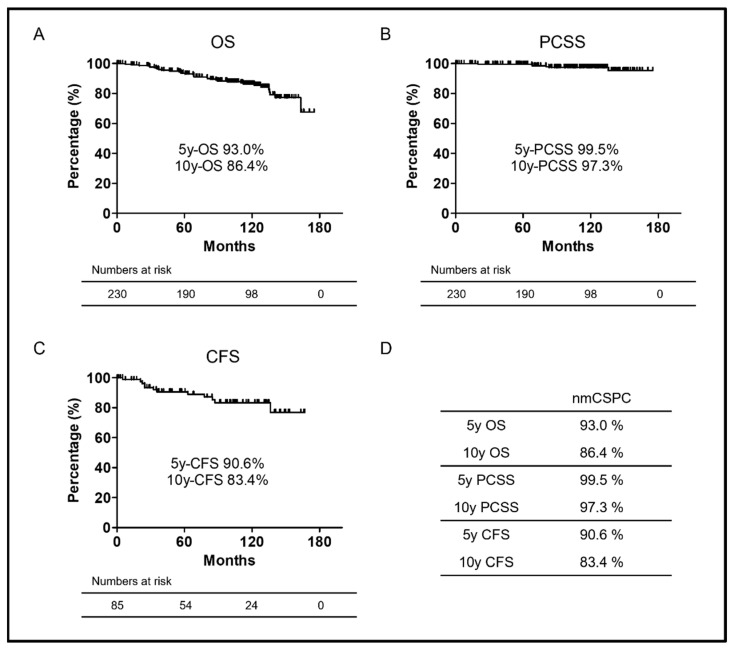
(**A**) Kaplan–Meier curves of OS in nmCSPC. (**B**) Kaplan–Meier curves of PCSS in nmCSPC. (**C**) Kaplan–Meier curves of CFS in in nmCSPC. (**D**) OS, PCSS, and CFS rate at five or 10 years in nmCSPC. Numbers at risk for each group are shown at the bottom of the figure. OS, overall survival; PCSS, prostate-cancer-specific survival; CFS, castration-resistant prostate-cancer-free survival; and nmCSPC, non-metastatic castration-sensitive prostate cancer.

Kaplan–Meier survival curves in the present cohort for OS, PCSS, and CFS are shown in Figure 1A–C. Since nmCSPC has a relatively good prognosis, the median OS, PCSS, and CFS were not all reached during this observation period. The five- and 10-year OS rates of the two groups were 93.0% and 86.4%, respectively (Figure 1D). The five- and 10-year PCSS rates of the two groups were 99.5% and 97.3%, respectively (Figure 1D). The five- and 10-year CFS rates of the two groups were 90.6% and 83.4%, respectively (Figure 1D).

### 3.5. Kaplan–Meier Curves of OS in nmCSPC Examined by Prognostic Factors

**Figure 2:**
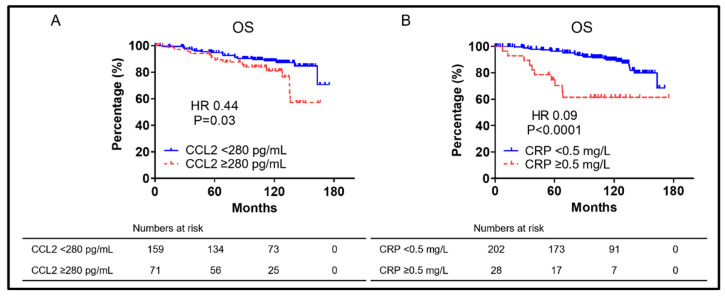
(**A**) Kaplan–Meier curves of OS in nmCSPC with CCL2 ≥ 280 pg/mL and CCL2 < 280 pg/mL. (**B**) Kaplan–Meier curves of OS in nmCSPC with CRP ≥ 0.5 mg/L and CRP < 0.5 mg/L. Numbers at risk for each group are shown at the bottom of the figure. OS, overall survival; CRP, C-reactive protein; HR, hazard ratio; and nmCSPC, non-metastatic castration-sensitive prostate cancer.

The OS for each factor that showed significant differences in the multivariate analysis in Table 2 is shown in Figure 2. Patients with CCL2 ≥ 280 pg/mL had significantly worse OS than those with CCL2 < 280 pg/mL (median OS: not reached vs. not reached, respectively; HR = 0.44; and *p* = 0.03) (Figure 2A). Patients with CRP ≥ 0.5 mg/L had significantly worse OS than those with CRP < 0.5 mg/L (median OS: not reached vs. not reached, respectively; HR = 0.09; and *p* < 0.0001) (Figure 2B).

### 3.6. Kaplan–Meier Curves of CFS in nmCSPC Examined by Prognostic Factors

**Figure 3:**
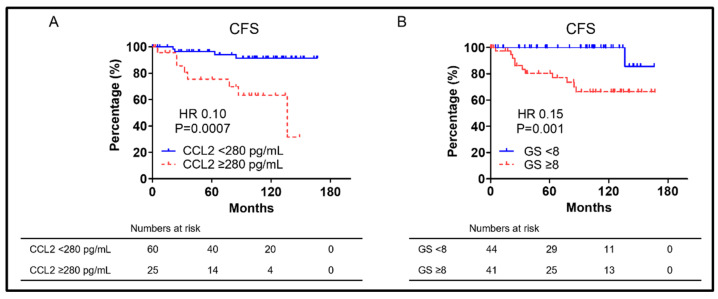
(**A**) Kaplan–Meier curves of CFS in nmCSPC with CCL2 ≥ 280 pg/mL and CCL2 < 280 pg/mL. (**B**) Kaplan–Meier curves of CFS in nmCSPC with GS ≥ 8 and GS < 8. Numbers at risk for each group are shown at the bottom of the figure. CFS, castration-resistant prostate-cancer-free survival; GS, Gleason score; HR, hazard ratio; and nmCSPC, non-metastatic castration-sensitive prostate cancer.

Each factor’s CFS showed significant multivariate analysis differences in Table 3 shown in Figure 3. Significantly worse CFS than those with CCL2 < 280 pg/mL (median CFS: 136.4 vs. not reached, respectively; HR = 0.10; and *p* = 0.0007) (Figure 3A) was demonstrated in patients with CCL2 ≥ 280 pg/mL. Significantly worse OS than those with GS < 8 (median OS: not reached vs. not reached, respectively; HR = 0.15; and *p* = 0.001) (Figure 3B) was demonstrated in patients with GS ≥ 8.

### 3.7. Kaplan–Meier Curves of MFS and VMFS in nmCSPC

**Figure 4:**
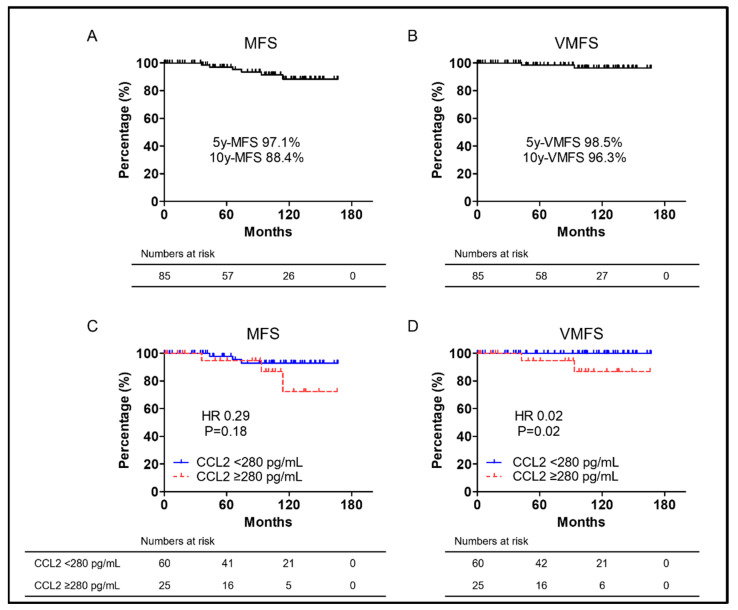
(**A**) Kaplan–Meier curves of MFS in nmCSPC. (**B**) Kaplan–Meier curves of VMFS in nmCSPC. (**C**) Kaplan–Meier curves of MFS in in nmCSPC with CCL2 ≥ 280 pg/mL and CCL2 < 280 pg/mL. (**D**) Kaplan–Meier curves of VMFS in in nmCSPC with CCL2 ≥ 280 pg/mL and CCL2 < 280 pg/mL. Numbers at risk for each group are shown at the bottom of the figure. MFS, metastasis-free survival; VMFS, visceral metastasis-free survival; HR, hazard ratio; and nmCSPC, non-metastatic castration-sensitive prostate cancer.

MFS is known as a prognostic nmCSPC indicator and the Kaplan–Meier curve for MFS is shown in Figure 4A. Median MFS was not reached. The five- and 10-year MFS rates were 97.1% and 88.4%, respectively. The VMFS Kaplan–Meier curve is also shown in Figure 4B. Median MFS was not reached. The five- and 10-year VMFS rates were 98.5% and 96.3%, respectively. Only six patients had metastasis after CRPC progression, of which only two patients had visceral metastasis. The number of events was so small that multivariate analysis was not performed. Figure 4C,D shows Kaplan–Meier curves of MFS and VMFS in the CCL2 ≥ 280 pg/mL and CCL2 < 280 pg/mL groups. Neither MFS nor VMFS reached the median There was no significant difference in MFS between patients with CCL2 ≥ 280 pg/mL and CCL2 < 280 pg/mL (median MFS: not reached vs. not reached, respectively; HR 0.29; and *p* = 0.18) (Figure 4C). Patients with CCL2 ≥ 280 pg/mL had significantly worse VMFS than those with CCL2 < 280 pg/mL (median VMFS: not reached vs. not reached, respectively; HR 0.02; and *p* = 0.02) (Figure 4D).

## 4. Discussion

CCL2 is also known as monocyte chemotaxis protein-1 (MCP-1) and was first discovered among CC chemokines in 1989 [20,21]. CCL2 is a 13 kDa protein composed of 76 amino acids. Its coding gene is mapped at human chromosome 17 (chr. 17, q11.2) [22,23] endothelial cells, epithelial cells, myeloid cells, smooth muscle cells, and fibroblasts are included in the variety of CCL2 expressions [24]. The biological CCL2 functions are mediated through its G-protein coupled receptor, C-C chemokine receptor 2 (CCR2) [25]. The CCL2 to CCR2 binding activates intracellular signaling cascades mediated by G proteins such as phosphatidylinositol 3-kinase (PI3K)/AKT, mitogen-activated protein kinase (MAPK)/p38, and Janus kinase (JAK)/STAT3 [26,27,28]. CCL2 is known to be associated with obesity, diabetes, cardiovascular disease, insensitivity, diabetic nephropathy, diabetic retinopathy, and other diseases [23,29,30]. CCL2 is reported to be associated with various cancers and has recently attracted much attention [23,31,32].

CCL2 is reported to directly induce PC cell line proliferation and migration in prostate cancer via phosphoinositide-3-kinase (PI3K)/serine-threonine kinase (Akt) signaling activation [16]. This led us to consider CCL2 as a potentially useful PC biomarker. We have measured serum CCL2 levels in 379 prostate biopsy patients and 255 patients were diagnosed with PC [17]. Patients with PC have been shown to have significantly higher CCL2 levels than patients without cancer [17]. This result is consistent with a pilot study that examined six chemokines as potential PC biomarkers [33]. Patients with CCL2 ≥ 320 pg/mL had significantly shorter OS, PCSS, and CFS than those with CCL2 < 320 pg/mL in our previous study [17]. Furthermore, in the long-term results with a median follow-up of 110.4 months, patients with CCL2 ≥ 320 pg/mL similarly had significantly shorter OS, PCSS, and CFS than those with CCL2 < 320 pg/mL [18]. A new risk classification was constructed using CCL2 ≥ 320 pg/mL, PSA ≥ 100 ng/mL, and GS ≥ 8 as risk factors, which was shown to predict OS compared to a single risk factor [18]. CCL2 was, therefore, considered to be a useful biomarker for determining PC patients’ prognosis, complementing PSA.

Many studies on CCL2′s role in prostate cancer have described migration capacity and metastasis. AR signaling suppression was previously demonstrated to inhibit PC cell proliferation and PSA secretion, was well as to promote CCL2 secretion, allowing PC cells to metastasize [27]. Anti-CCL2 monoclonal antibodies also markedly inhibited the prostate-cancer bone metastasis progression in intracardiac and intra-tibial models [34]. The interaction between CCL2 and CCR2 was reported to promote prostate-cancer cell migration via increased αvβ3 integrin production [35]. Signaling from WNT5A via CCL2 and bone morphogenetic protein 6 (BMP6) was reported to induce CRPC [36]. CCL2 was speculated to be a CFS and MFS predictor. This study focused on nmCSPC cases based on the above. CCL2 ≥ 280 pg/mL was a prognostic OS and CFS predictor as shown in Figure 2 and Figure 3. The cut-off CCL2 value was set at 320 pg/mL, but CCL2 has been found to increase with prostate-cancer progression in the prior paper [17,18]. In this study, the cut-off CCL2 value was set at 280 pg/mL as it was the value that most clearly separated the two groups, which is limited to nmCSPC. There was no significant difference in MFS between patients with CCL2 ≥ 280 pg/mL and those with CCL2 < 280 pg/mL, although multivariate analysis was not performed due to the small number of events in the six patients who developed metastasis after CRPC progression. However, patients with CCL2 ≥ 280 pg/mL had significantly shorter VMFS than those with CCL2 < 280 pg/mL when restricted to patients who developed visceral metastasis as shown in Figure 4A. Targeting PC AR with siRNA promoted PC cell migration and metastasis via CCL2-dependent STAT3 activation and epithelial–mesenchymal transition (EMT) pathways was previously revealed [27]. High CCL2 levels may indicate a small AR signal dependence percentage. A larger study could show a significant difference, although the number of events in this study was small and no significant difference was found in MFS. It is assumed that a significant difference was found for VMFS because no patient in the group with CCL2 < 280 developed visceral metastases. A strong AR suppression signaling increases visceral metastases was previously shown, although visceral metastases are rare in prostate cancer [2]. The risk of developing visceral metastases may be very low in the group with low CCL2 levels at the time of prostate-cancer diagnosis. CCL2 is not only a predictor of OS and CFS in this study, but it may also predict metastasis.

We examined CCL2 levels in a wild-type PC cell line (DU145), a DTX-resistant PC cell line (DU145-TxR), and a CBZ-resistant PC cell line (DU145 TxR/CxR), and found that DU145-TxR and DU145 TxR/CxR had significantly higher CCL2 levels than DU145 [37]. CCL2 treatment of DU145 resulted in CBZ resistance, and CCR2 (specific receptor for CCL2) antagonist treatment of DU145-TxR/CxR was also shown to mitigate CBZ resistance [37]. In addition, CCL2-mediated DTX resistance has been reported. It has been shown that CCL2 activity inhibition has anti-tumor effects, and that the combination of CCL2 and DTX enhances the therapeutic DTX effect [38,39]. CCL2 may be a predictive biomarker of response to taxane-based chemotherapy, although further studies are needed. CCL2 is associated with radioresistance. There are reports that CCL2 inhibition improves radiosensitivity, so CCL2 may also be a biomarker when considering radiotherapy indication in localized prostate cancers [40]. Serum CCL2 may assist in the treatment selection for nmCSPC.

CRP was a predictor of OS in nmCSPC in this study. Inflammation is associated with the etiology and progression of solid tumors [41]. CRP is one of the most sensitive and easily measured factors in the inflammatory response. CRP has been associated with a variety of malignancies, including prostate, esophageal, kidney, and ovarian cancers [42,43,44,45,46]. High CRP levels were reported to be an OS and PCS predictor in prostate-cancer patients, which is consistent with the present study in a meta-analysis [42,47,48]. CRP may be useful as a complementary biomarker to PSA, but has not been reported as a predictor of CFS or MFS at all. CCL2 may be a more useful biomarker for nmCSPC than CRP, since CCL2 was a predictor of CFS in this study.

There are several limitations in the current study. This was a retrospective study with a relatively small number of all-Japanese patients. In addition, PC treatment and the interval between imaging assessment are at the attending physician’s discretion. A larger prospective study is needed to confirm the results of this study.

## 5. Conclusions

This study is the first to demonstrate the serum CCL2 utility as a biomarker to predict OS and CFS in nmCSPC. Serum CCL2 may help in the selection of treatment for nmCSPC, although further studies are warranted.

## Figures and Tables

**Table 1:** Patients’ characteristics.

Characteristics	Value
Patients, *n*	230
Median age at diagnosis of PC, y (range)	69 (50–89)
Median PSA at diagnosis of PC, ng/mL (range)	9.4 (1.5–227.8)
Median CCL2 at diagnosis of PC, pg/mL (range)	244.5 (95.3–749)
Histology	
GS ≤ 6	59
GS = 7	97
GS ≥ 8	74
T stage at diagnosis of PC	
T1	60
T2	128
≥T3	42
N stage at diagnosis of PC	
N0	218
N1	12
Primary localized treatment	
ADT only	63
Radiation ± ADT	127
RP ± ADT	35
AS	5
Patients who progressed to CRPC, n	12
Patients who progressed to mCRPC, n	6
All-cause death, n	34
PC-specific death, n	6

PC = prostate cancer; PSA = prostate-specific antigen; GS = Gleason score; ADT = androgen-deprivation therapy; RP = radical prostatectomy; AS = active surveillance; CRPC = castration-resistant prostate cancer; and mCRPC = metastatic CRPC.

**Table 2:** Univariate and multivariate analyses of OS.

			Univariate		Multivariate	
		n	HR (95% CI)	*p*	HR (95% CI)	*p*
Age at diagnosis, y	<65	65	2.32 (0.96–5.61)	0.06		
	≥65	165				
T stage	≤T2	188	1.90 (0.88–4.06)	0.1		
	≥T3	42				
N stage	0	218	3.49 (1.34–9.11)	0.01	2.36 (0.69–8.10)	0.17
	1	12				
PSA, ng/mL	<20	182	2.25 (1.10–4.63)	0.03	2.00 (0.74–5.40)	0.17
	≥20	48				
Gleason score	≤7	156	2.07 (1.06–4.07)	0.03	0.96 (0.42–2.23)	0.93
	≥8	74				
CCL2, pg/mL	<280	159	2.07 (1.05–4.07)	0.04	2.31 (1.11–4.81)	0.03
	≥280	71				
CRP, mg/dL	<0.5	202	5.30 (2.34–11.99)	<0.001	4.89 (2.12–11.28)	<0.001
	≥0.5	21				
ALP, IU/L	<350	214	3.14 (0.94–10.44)	0.06		
	≥350	12				

OS = overall survival; PSA = prostate-specific antigen; CCL2 = serum chemokine ligand 2; CRP = C-reactive protein; and ALP = alkaline phosphatase.

**Table 3:** Univariate and multivariate analyses of CFS.

			Univariate		Multivariate	
		n	HR (95% CI)	*p*	HR (95% CI)	*p*
Age at diagnosis, y	<65	15	0.76 (0.21–2.83)	0.69		
	≥65	70				
T stage	≤T2	57	2.28 (0.73–7.07)	0.15		
	≥T3	28				
N stage	0	74	6.19 (1.96–19.60)	0.002	2.37 (0.71–7.94)	0.16
	1	11				
PSA, ng/mL	<20	53	2.89 (0.92–9.11)	0.07		
	≥20	32				
Gleason score	≤7	51	13.43 (1.73–104.09)	0.01	8.54 (1.03–70.91)	0.047
	≥8	34				
CCL2, pg/mL	<280	60	6.21 (1.86–20.73)	0.003	4.50 (1.20–16.86)	0.03
	≥280	25				
CRP, mg/dL	<0.5	71	2.71 (0.57–13.04)	0.21		
	≥0.5	9				
ALP, IU/L	<350	74	2.38 (0.50–11.31)	0.28		
	≥350	9				

CFS = castration-resistant prostate-cancer-free survival; PSA = prostate-specific antigen; CCL2 = serum chemokine ligand 2; CRP = C-reactive protein; and ALP = alkaline phosphatase.

## Data Availability

The comprehensive study protocol is available from the authors. Data can be provided by the authors upon request.
